# Supplementary material for: Characterizing the spatial patterns and determinants of cerebrospinal fluid pseudorandom flow in the human brain with low b-value diffusion MRI
Source: Imaging Neurosci (Camb). 2025 Feb 18;3:imag_a_00473. doi: 10.1162/imag_a_00473 (PMC12048033; doi:10.1162/imag_a_00473)
Supplement: Supplementary Material [file imag_a_00473-supp.pdf]

Characterizing the Spatial Patterns and Determinants of Cerebrospinal Fluid Pseudorandom Flow in the Human Brain with Low b-value Diffusion MRI – Supplementary Material

Arash Nazeri[1*#], Helia Hosseini[1], Taher Dehkharghanian[2], Kevin E. Lindsay[1], Pamela LaMontagne[1], Joshua S. Shimony[1], Tammie L.S. Benzinger[1,3], Aristeidis Sotiras[1,4*]

[1]Mallinckrodt Institute of Radiology, Washington University School of Medicine, St. Louis, MO, USA

[2]University Health Network, Toronto, Ontario, Canada

[3]Knight Alzheimer's Disease Research Center, Washington University School of Medicine, St. Louis, MO, USA

[4]Institute of Informatics, Washington University School of Medicine, St. Louis, MO, USA

[*]Correspondence: a.nazeri@wustl.edu (A.N.); aristeidis.sotiras@wustl.edu (A.S.)
[#]The Biomedical MR Center (BMRC), Mallinckrodt Institute of Radiology, Washington University School of Medicine, 660 S. Euclid Ave., MSC 8131-0050-05, St. Louis, Missouri 63110.

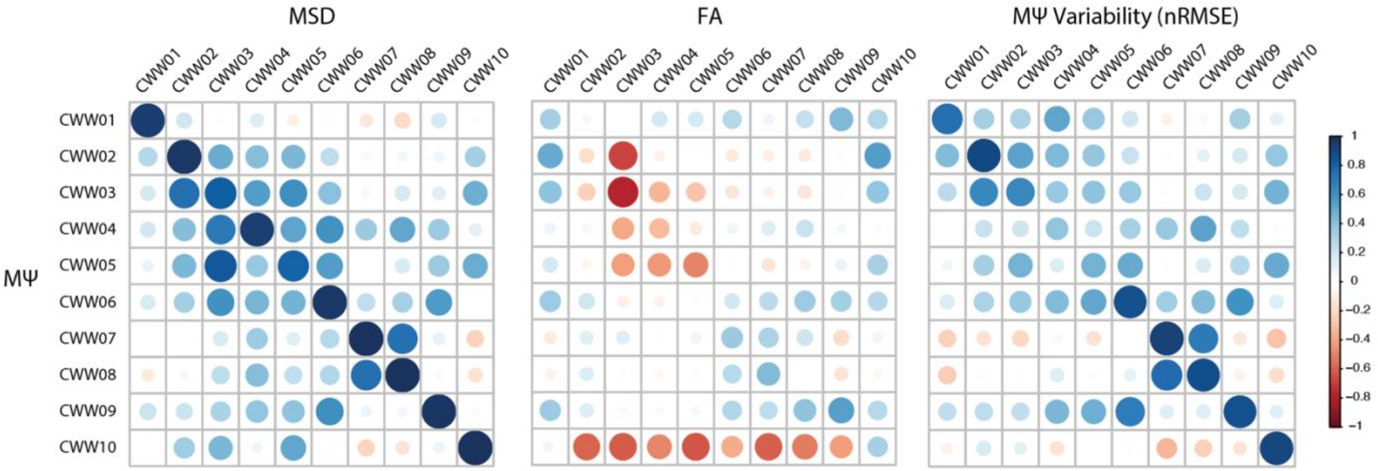

Figure S1. Correlation between MΨ and other CSF flow metrics derived from low b-value dMRI across CWWs in the CDMD dataset.

Abbreviations: CWWs, CSF waterways; dMRI: diffusion-weighted MRI; MΨ, mean pseudo-diffusivity.

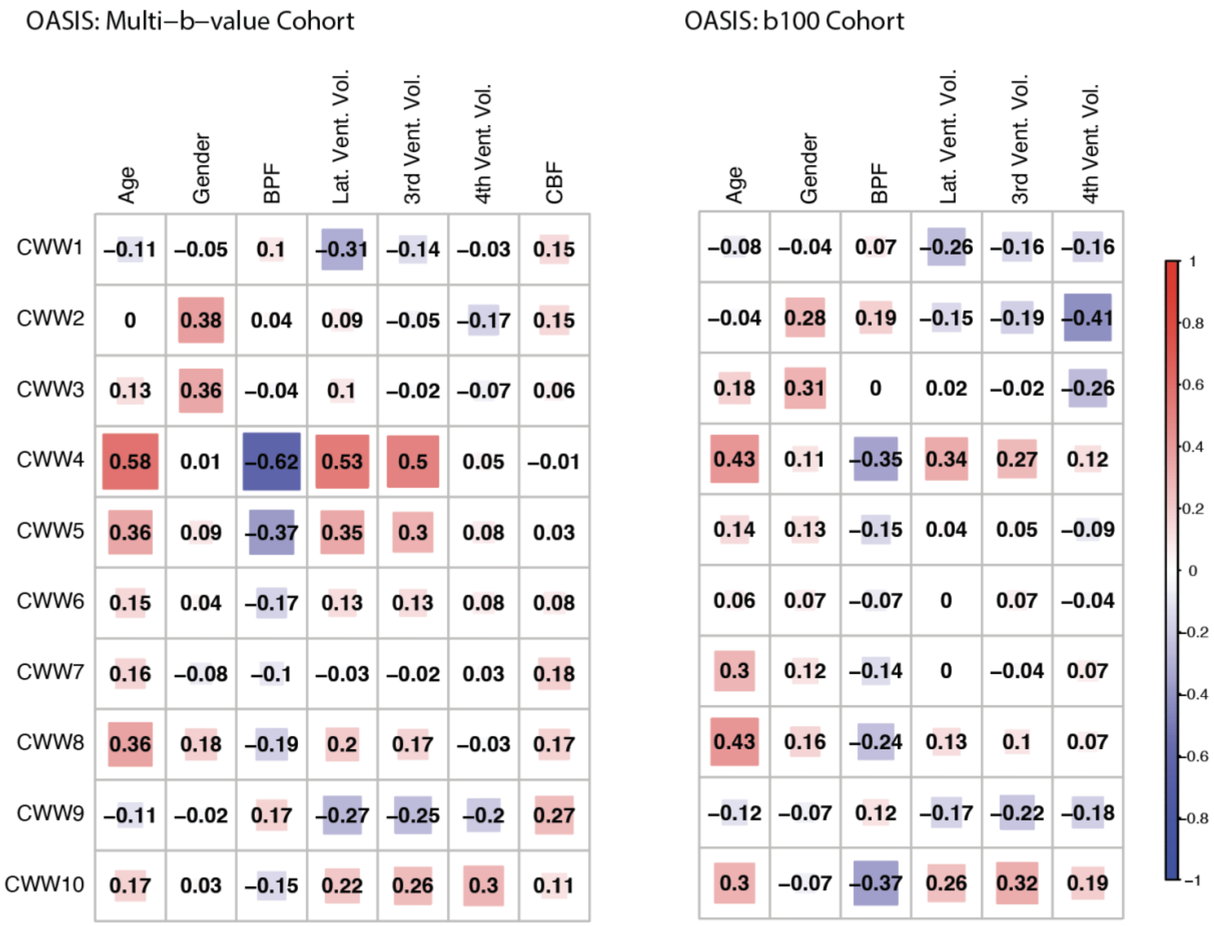

Figure S2. Unadjusted bivariate correlations between CWW MΨ and demographic variables, intracranial anatomy, and cerebral perfusion.

Abbreviations: BPF, brain parenchymal fraction; CBF, cerebral blood flow; CWWs, CSF waterways; MΨ, mean pseudo-diffusivity.
